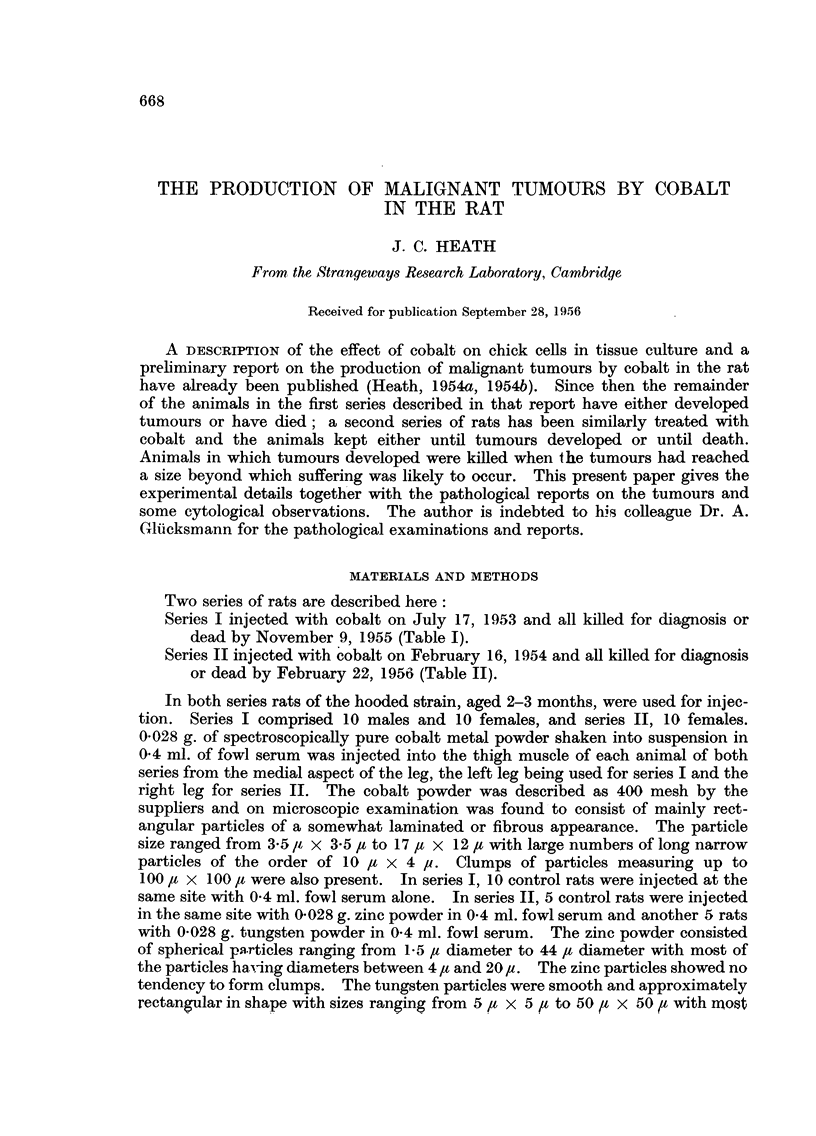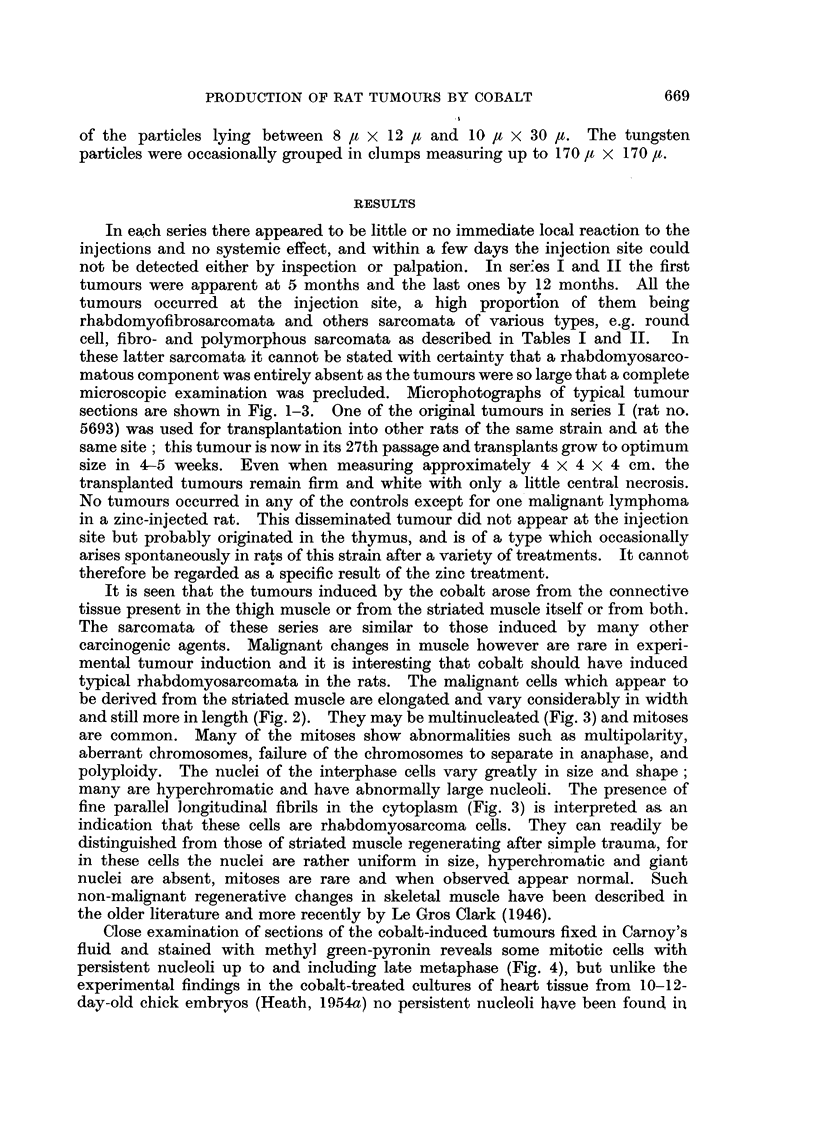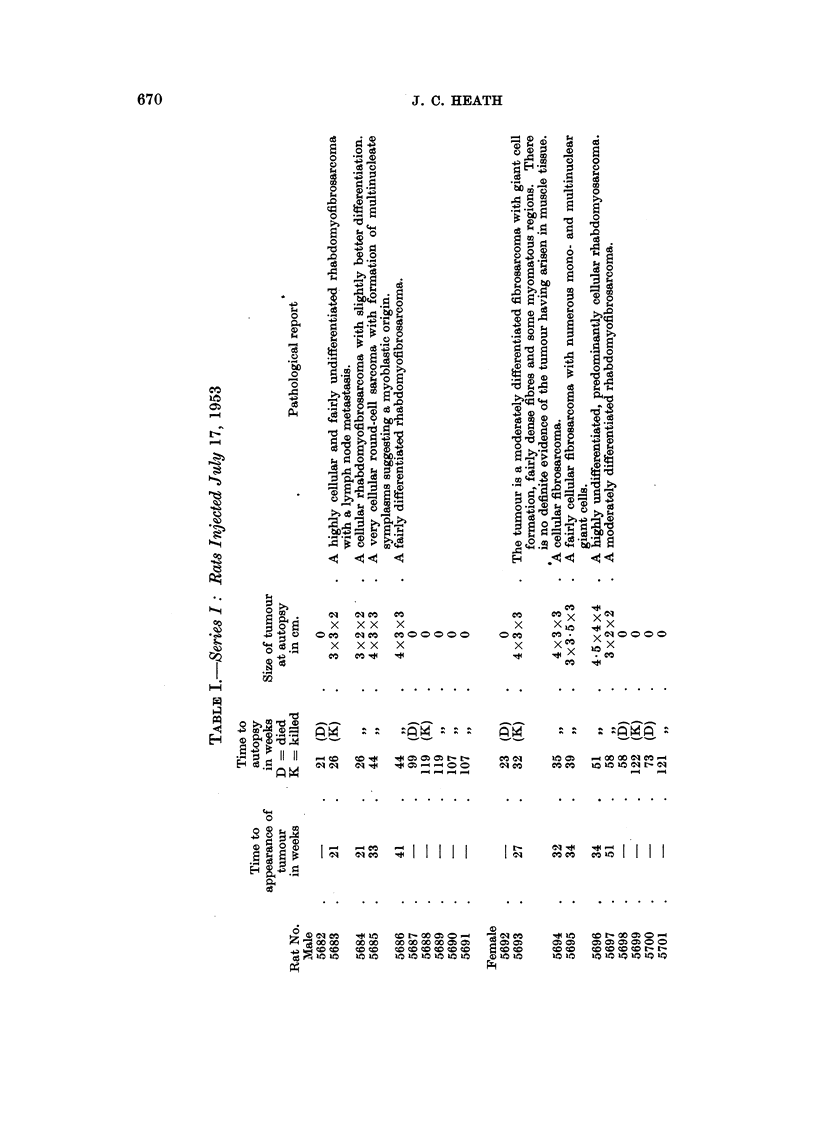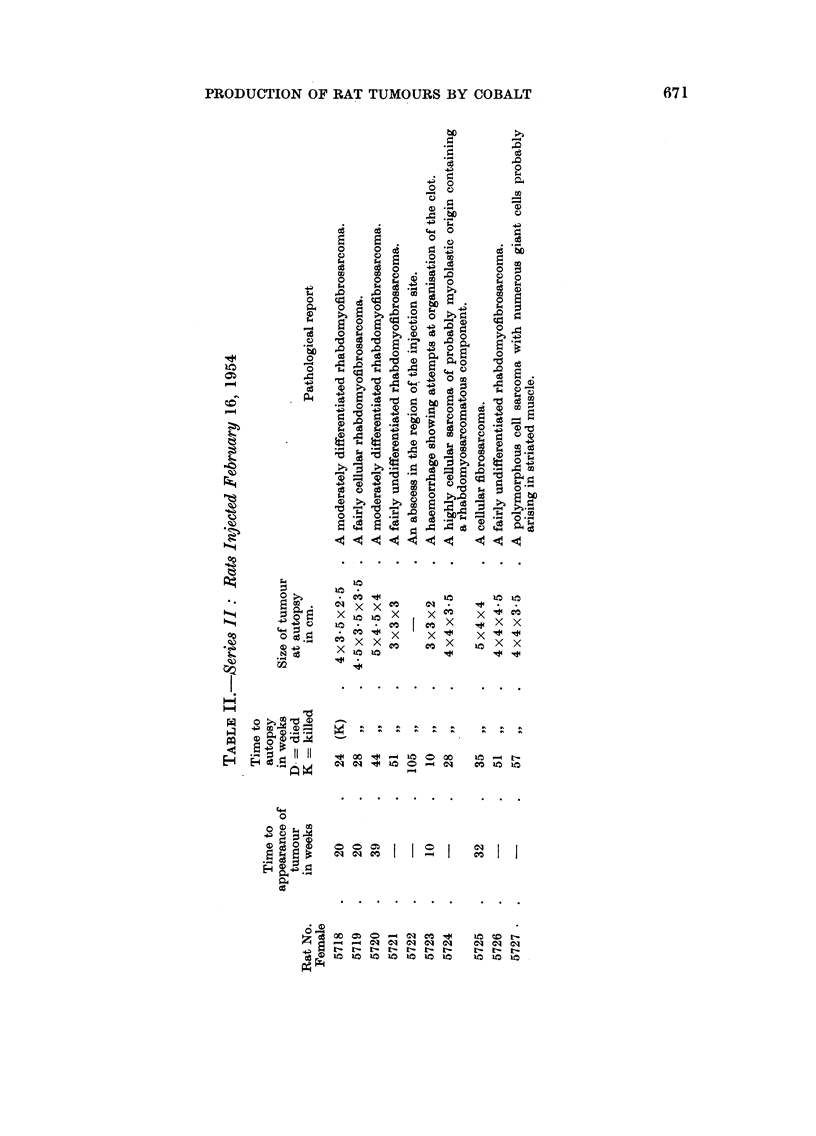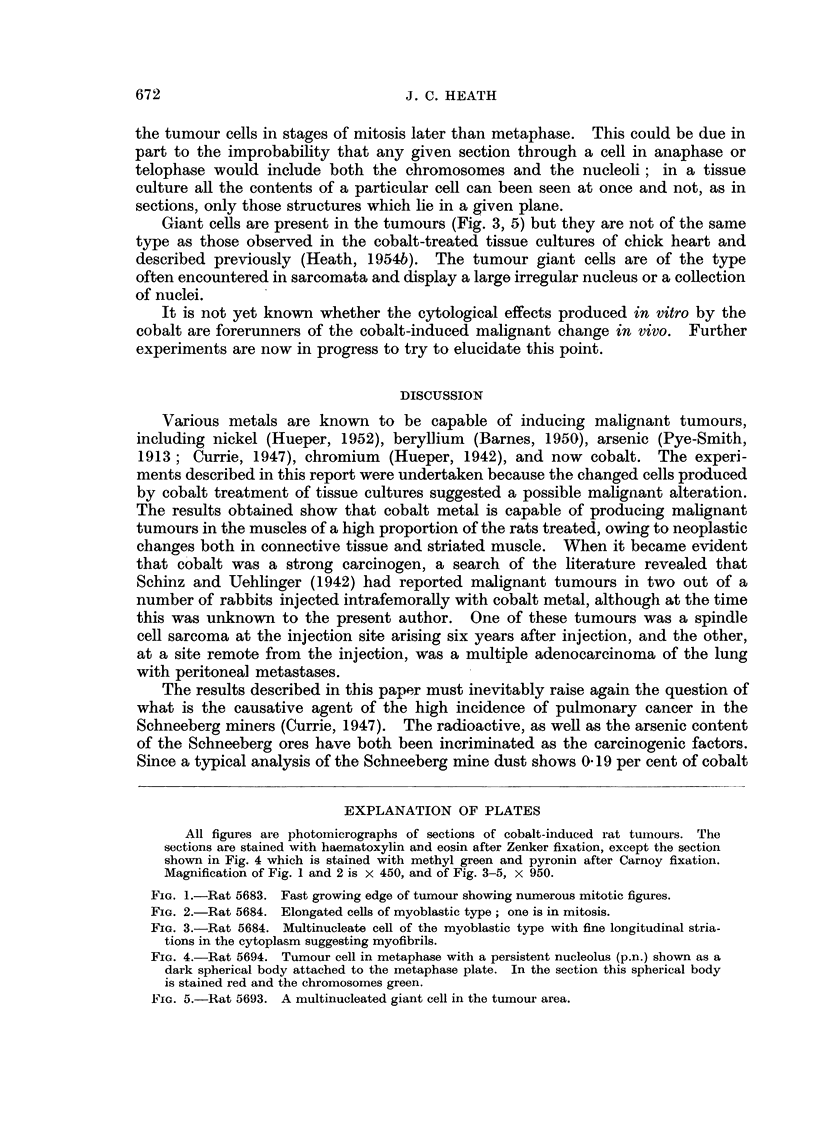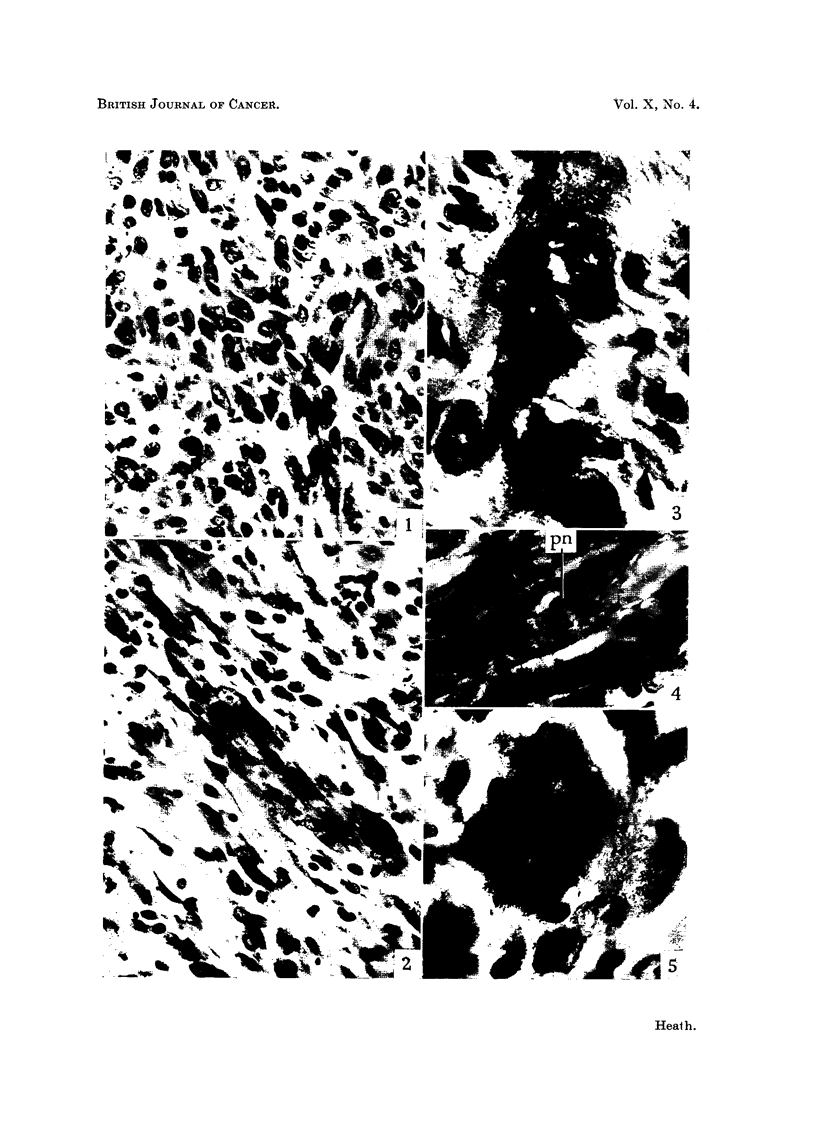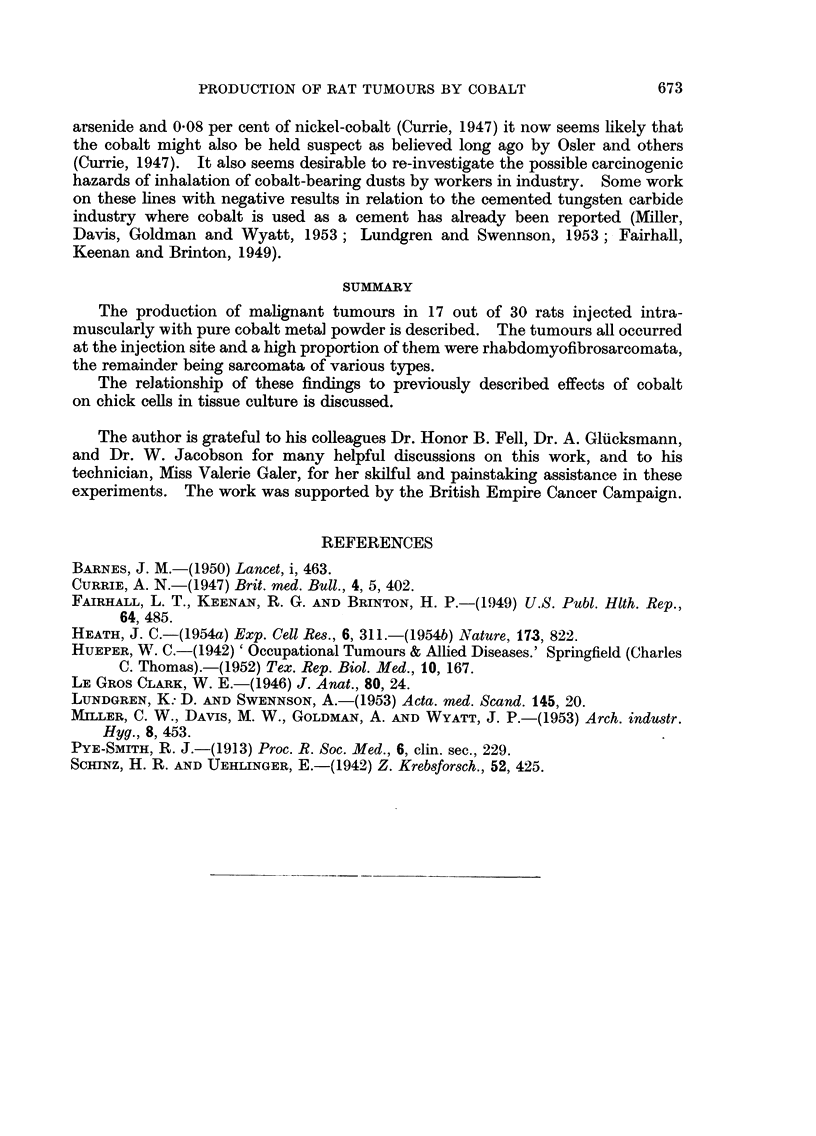# The Production of Malignant Tumours by Cobalt in the Rat

**DOI:** 10.1038/bjc.1956.80

**Published:** 1956-12

**Authors:** J. C. Heath

## Abstract

**Images:**


### THE PRODUCTION OF MALIGNANT TUMOURS BY COBALT IN THE RAT

J. C. HEATH

*From the Strangeways Research Laboratory, Cambridge*

Received for publication September 28, 1956

A DESCRIPTION of the effect of cobalt on chick cells in tissue culture and a preliminary report on the production of malignant tumours by cobalt in the rat have already been published (Heath, 1954*a*, 1954*b*). Since then the remainder of the animals in the first series described in that report have either developed tumours or have died ; a second series of rats has been similarly treated with cobalt and the animals kept either until tumours developed or until death. Animals in which tumours developed were killed when the tumours had reached a size beyond which suffering was likely to occur. This present paper gives the experimental details together with the pathological reports on the tumours and some cytological observations. The author is indebted to his colleague Dr. A. Glücksmann for the pathological examinations and reports.

### MATERIALS AND METHODS

Two series of rats are described here :

Series I injected with cobalt on July 17, 1953 and all killed for diagnosis or dead by November 9, 1955 (Table I).

Series II injected with cobalt on February 16, 1954 and all killed for diagnosis or dead by February 22, 1956 (Table II).

In both series rats of the hooded strain, aged 2–3 months, were used for injection. Series I comprised 10 males and 10 females, and series II, 10 females. 0·028 g. of spectroscopically pure cobalt metal powder shaken into suspension in 0·4 ml. of fowl serum was injected into the thigh muscle of each animal of both series from the medial aspect of the leg, the left leg being used for series I and the right leg for series II. The cobalt powder was described as 400 mesh by the suppliers and on microscopic examination was found to consist of mainly rectangular particles of a somewhat laminated or fibrous appearance. The particle size ranged from 3·5 $\mu \times$ 3·5 $\mu$ to 17 $\mu \times$ 12 $\mu$ with large numbers of long narrow particles of the order of 10 $\mu \times$ 4 $\mu$. Clumps of particles measuring up to 100 $\mu \times$ 100 $\mu$ were also present. In series I, 10 control rats were injected at the same site with 0·4 ml. fowl serum alone. In series II, 5 control rats were injected in the same site with 0·028 g. zinc powder in 0·4 ml. fowl serum and another 5 rats with 0·028 g. tungsten powder in 0·4 ml. fowl serum. The zinc powder consisted of spherical particles ranging from 1·5 $\mu$ diameter to 44 $\mu$ diameter with most of the particles having diameters between 4 $\mu$ and 20 $\mu$. The zinc particles showed no tendency to form clumps. The tungsten particles were smooth and approximately rectangular in shape with sizes ranging from 5 $\mu \times$ 5 $\mu$ to 50 $\mu \times$ 50 $\mu$ with most of the particles lying between 8 $\mu \times 12$ $\mu$ and 10 $\mu \times 30$ $\mu$. The tungsten particles were occasionally grouped in clumps measuring up to 170 $\mu \times 170$ $\mu$.

### RESULTS

In each series there appeared to be little or no immediate local reaction to the injections and no systemic effect, and within a few days the injection site could not be detected either by inspection or palpation. In series I and II the first tumours were apparent at 5 months and the last ones by 12 months. All the tumours occurred at the injection site, a high proportion of them being rhabdomyofibrosarcomata and others sarcomata of various types, e.g. round cell, fibro- and polymorphous sarcomata as described in Tables I and II. In these latter sarcomata it cannot be stated with certainty that a rhabdomyosarcomatous component was entirely absent as the tumours were so large that a complete microscopic examination was precluded. Microphotographs of typical tumour sections are shown in Fig. 1–3. One of the original tumours in series I (rat no. 5693) was used for transplantation into other rats of the same strain and at the same site ; this tumour is now in its 27th passage and transplants grow to optimum size in 4–5 weeks. Even when measuring approximately 4 × 4 × 4 cm. the transplanted tumours remain firm and white with only a little central necrosis. No tumours occurred in any of the controls except for one malignant lymphoma in a zinc-injected rat. This disseminated tumour did not appear at the injection site but probably originated in the thymus, and is of a type which occasionally arises spontaneously in rats of this strain after a variety of treatments. It cannot therefore be regarded as a specific result of the zinc treatment.

It is seen that the tumours induced by the cobalt arose from the connective tissue present in the thigh muscle or from the striated muscle itself or from both. The sarcomata of these series are similar to those induced by many other carcinogenic agents. Malignant changes in muscle however are rare in experimental tumour induction and it is interesting that cobalt should have induced typical rhabdomyosarcomata in the rats. The malignant cells which appear to be derived from the striated muscle are elongated and vary considerably in width and still more in length (Fig. 2). They may be multinucleated (Fig. 3) and mitoses are common. Many of the mitoses show abnormalities such as multipolarity, aberrant chromosomes, failure of the chromosomes to separate in anaphase, and polyploidy. The nuclei of the interphase cells vary greatly in size and shape ; many are hyperchromatic and have abnormally large nucleoli. The presence of fine parallel longitudinal fibrils in the cytoplasm (Fig. 3) is interpreted as an indication that these cells are rhabdomyosarcoma cells. They can readily be distinguished from those of striated muscle regenerating after simple trauma, for in these cells the nuclei are rather uniform in size, hyperchromatic and giant nuclei are absent, mitoses are rare and when observed appear normal. Such non-malignant regenerative changes in skeletal muscle have been described in the older literature and more recently by Le Gros Clark (1946).

Close examination of sections of the cobalt-induced tumours fixed in Carnoy's fluid and stained with methyl green-pyronin reveals some mitotic cells with persistent nucleoli up to and including late metaphase (Fig. 4), but unlike the experimental findings in the cobalt-treated cultures of heart tissue from 10–12-day-old chick embryos (Heath, 1954*a*) no persistent nucleoli have been found in

TABLE I.—*Series I : Rats Injected July* 17, 1953

| Rat No. | Time to appearance of tumour in weeks | Time to autopsy in weeks D = died K = killed | Size of tumour at autopsy in cm. | Pathological report |
|---|---|---|---|---|
| Male | | | | |
| 5682 | — | 21 (D) | 0 | |
| 5683 | 21 | 26 (K) | 3×3×2 | A highly cellular and fairly undifferentiated rhabdomyofibrosarcoma with a lymph node metastasis. |
| 5684 | 21 | 26 „ | 3×2×2 | A cellular rhabdomyofibrosarcoma with slightly better differentiation. |
| 5685 | 33 | 44 „ | 4×3×3 | A very cellular round-cell sarcoma with formation of multinucleate symplasms suggesting a myoblastic origin. |
| 5686 | 41 | 44 „ | 4×3×3 | A fairly differentiated rhabdomyofibrosarcoma. |
| 5687 | — | 99 (D) | 0 | |
| 5688 | — | 119 (K) | 0 | |
| 5689 | — | 119 „ | 0 | |
| 5690 | — | 107 „ | 0 | |
| 5691 | — | 107 „ | 0 | |
| Female | | | | |
| 5692 | — | 23 (D) | 0 | |
| 5693 | 27 | 32 (K) | 4×3×3 | The tumour is a moderately differentiated fibrosarcoma with giant cell formation, fairly dense fibres and some myomatous regions. There is no definite evidence of the tumour having arisen in muscle tissue. |
| 5694 | 32 | 35 „ | 4×3×3 | A cellular fibrosarcoma. |
| 5695 | 34 | 39 „ | 3×3·5×3 | A fairly cellular fibrosarcoma with numerous mono- and multinuclear giant cells. |
| 5696 | 34 | 51 „ | 4·5×4×4 | A highly undifferentiated, predominantly cellular rhabdomyosarcoma. |
| 5697 | 51 | 58 „ | 3×2×2 | A moderately differentiated rhabdomyofibrosarcoma. |
| 5698 | — | 58 (D) | 0 | |
| 5699 | — | 122 (K) | 0 | |
| 5700 | — | 73 (D) | 0 | |
| 5701 | — | 121 „ | 0 | |

TABLE II.—*Series II : Rats Injected February* 16, 1954

| Rat No. Female | Time to appearance of tumour in weeks | Time to autopsy in weeks D = died K = killed | Size of tumour at autopsy in cm. | Pathological report |
|---|---|---|---|---|
| 5718 | 20 | 24 (K) | 4×3·5×2·5 | A moderately differentiated rhabdomyofibrosarcoma. |
| 5719 | 20 | 28 „ | 4·5×3·5×3·5 | A fairly cellular rhabdomyofibrosarcoma. |
| 5720 | 39 | 44 „ | 5×4·5×4 | A moderately differentiated rhabdomyofibrosarcoma. |
| 5721 | — | 51 „ | 3×3×3 | A fairly undifferentiated rhabdomyofibrosarcoma. |
| 5722 | — | 105 „ | — | An abscess in the region of the injection site. |
| 5723 | 10 | 10 „ | 3×3×2 | A haemorrhage showing attempts at organisation of the clot. |
| 5724 | — | 28 „ | 4×4×3·5 | A highly cellular sarcoma of probably myoblastic origin containing a rhabdomyosarcomatous component. |
| 5725 | 32 | 35 „ | 5×4×4 | A cellular fibrosarcoma. |
| 5726 | — | 51 „ | 4×4×4·5 | A fairly undifferentiated rhabdomyofibrosarcoma. |
| 5727 | — | 57 „ | 4×4×3·5 | A polymorphous cell sarcoma with numerous giant cells probably arising in striated muscle. |

the tumour cells in stages of mitosis later than metaphase. This could be due in part to the improbability that any given section through a cell in anaphase or telophase would include both the chromosomes and the nucleoli ; in a tissue culture all the contents of a particular cell can been seen at once and not, as in sections, only those structures which lie in a given plane.

Giant cells are present in the tumours (Fig. 3, 5) but they are not of the same type as those observed in the cobalt-treated tissue cultures of chick heart and described previously (Heath, 1954*b*). The tumour giant cells are of the type often encountered in sarcomata and display a large irregular nucleus or a collection of nuclei.

It is not yet known whether the cytological effects produced *in vitro* by the cobalt are forerunners of the cobalt-induced malignant change *in vivo*. Further experiments are now in progress to try to elucidate this point.

### DISCUSSION

Various metals are known to be capable of inducing malignant tumours, including nickel (Hueper, 1952), beryllium (Barnes, 1950), arsenic (Pye-Smith, 1913 ; Currie, 1947), chromium (Hueper, 1942), and now cobalt. The experiments described in this report were undertaken because the changed cells produced by cobalt treatment of tissue cultures suggested a possible malignant alteration. The results obtained show that cobalt metal is capable of producing malignant tumours in the muscles of a high proportion of the rats treated, owing to neoplastic changes both in connective tissue and striated muscle. When it became evident that cobalt was a strong carcinogen, a search of the literature revealed that Schinz and Uehlinger (1942) had reported malignant tumours in two out of a number of rabbits injected intrafemorally with cobalt metal, although at the time this was unknown to the present author. One of these tumours was a spindle cell sarcoma at the injection site arising six years after injection, and the other, at a site remote from the injection, was a multiple adenocarcinoma of the lung with peritoneal metastases.

The results described in this paper must inevitably raise again the question of what is the causative agent of the high incidence of pulmonary cancer in the Schneeberg miners (Currie, 1947). The radioactive, as well as the arsenic content of the Schneeberg ores have both been incriminated as the carcinogenic factors. Since a typical analysis of the Schneeberg mine dust shows 0·19 per cent of cobalt

---

### EXPLANATION OF PLATES

All figures are photomicrographs of sections of cobalt-induced rat tumours. The sections are stained with haematoxylin and eosin after Zenker fixation, except the section shown in Fig. 4 which is stained with methyl green and pyronin after Carnoy fixation. Magnification of Fig. 1 and 2 is × 450, and of Fig. 3–5, × 950.

FIG. 1.—Rat 5683. Fast growing edge of tumour showing numerous mitotic figures.

FIG. 2.—Rat 5684. Elongated cells of myoblastic type ; one is in mitosis.

FIG. 3.—Rat 5684. Multinucleate cell of the myoblastic type with fine longitudinal striations in the cytoplasm suggesting myofibrils.

FIG. 4.—Rat 5694. Tumour cell in metaphase with a persistent nucleolus (p.n.) shown as a dark spherical body attached to the metaphase plate. In the section this spherical body is stained red and the chromosomes green.

FIG. 5.—Rat 5693. A multinucleated giant cell in the tumour area.

Heath.

arsenide and 0·08 per cent of nickel-cobalt (Currie, 1947) it now seems likely that the cobalt might also be held suspect as believed long ago by Osler and others (Currie, 1947). It also seems desirable to re-investigate the possible carcinogenic hazards of inhalation of cobalt-bearing dusts by workers in industry. Some work on these lines with negative results in relation to the cemented tungsten carbide industry where cobalt is used as a cement has already been reported (Miller, Davis, Goldman and Wyatt, 1953; Lundgren and Swennson, 1953; Fairhall, Keenan and Brinton, 1949).

SUMMARY

The production of malignant tumours in 17 out of 30 rats injected intramuscularly with pure cobalt metal powder is described. The tumours all occurred at the injection site and a high proportion of them were rhabdomyofibrosarcomata, the remainder being sarcomata of various types.

The relationship of these findings to previously described effects of cobalt on chick cells in tissue culture is discussed.

The author is grateful to his colleagues Dr. Honor B. Fell, Dr. A. Glücksmann, and Dr. W. Jacobson for many helpful discussions on this work, and to his technician, Miss Valerie Galer, for her skilful and painstaking assistance in these experiments. The work was supported by the British Empire Cancer Campaign.

### REFERENCES

Barnes, J. M.—(1950) *Lancet*, i, 463.
Currie, A. N.—(1947) *Brit. med. Bull.*, **4**, 5, 402.
Fairhall, L. T., Keenan, R. G. and Brinton, H. P.—(1949) *U.S. Publ. Hlth. Rep.*, **64**, 485.
Heath, J. C.—(1954*a*) *Exp. Cell Res.*, **6**, 311.—(1954*b*) *Nature*, **173**, 822.
Hueper, W. C.—(1942) 'Occupational Tumours & Allied Diseases.' Springfield (Charles C. Thomas).—(1952) *Tex. Rep. Biol. Med.*, **10**, 167.
Le Gros Clark, W. E.—(1946) *J. Anat.*, **80**, 24.
Lundgren, K. D. and Swennson, A.—(1953) *Acta. med. Scand.* **145**, 20.
Miller, C. W., Davis, M. W., Goldman, A. and Wyatt, J. P.—(1953) *Arch. industr. Hyg.*, **8**, 453.
Pye-Smith, R. J.—(1913) *Proc. R. Soc. Med.*, **6**, clin. sec., 229.
Schinz, H. R. and Uehlinger, E.—(1942) *Z. Krebsforsch.*, **52**, 425.